# Important Developments Foreshadowed

**Published:** 1919-01-25

**Authors:** 


### A YEAR'S WORK AT THE LEICESTER ROYAL INFIRMARY.

### Important Developments Foreshadowed.

At a quarterly meeting of the governors and subscribers of the Leicester Royal Infirmary held on January 18, Mr. T. Fielding Johnson, junr. (High Sheriff), presented a report on the past year's work. The number of in-patients to the civilian wards (3,565) showed an increase of 253 on the previous year. Military patients numbered 452, a decline of 445, the total admissions thus being 4,010 as compared with 4,409. The greatly increased work of the out-patients' department reflected to some extent the response to the country's call by civil practitioners. A total of 28,394 attendances was recorded, as compared with 19,927, an increase of approximately 42 per cent.

### The Postponed Extensions.

The orthopædic department recorded 15,524 attendances, as compared with about 6,000 in 1917. In this department Dr. Astley Clarke had done excellent work for discharged soldiers as well as for civilian patients, but continued to be much handicapped by want of adequate accommodation. The board had made efforts to remedy this state of things by seeking permission to build and equip a separate and self-contained orthopædic department. They had been encouraged to do so by the Ministry of Pensions, but had been deprived of the means by which such a department could be established. So fully did the board realise the need of such a department that they had instructed the architect to prepare the necessary plans, and they were led to hope that, if facilities were provided for the treatment of discharged soldiers, a small grant-in-aid might be received from the Ministry of Pensions. Such a department would be a great asset to the community, and would, to an extent, bring the advantages of hydropathic centres to aid their recovery.

The proposed pathological department and mortuary, for which foundations were being made at the outbreak of war, would immediately be proceeded with, and the architect was now issuing tenders for the structure. The estimated pre-war cost was about £5,000. The growing importance of pathology in the treatment of sickness and disease, and the inadequacy of the present mortuary, made the department the first item in the progressive scheme of development which the board had under consideration, with a view of maintaining the equipment and service of the institution in a high state of efficiency.

The memorial wing to Sir Edward Wood would, now that the war was over, be taken into consideration, and the governors might rest assured that the final plan would fully maintain the standard set by Sir Edward Wood for beautiful hospital buildings. Roughly one-half of the cost had been paid and promised, and it would be a memorable gift if it might be recorded in Leicester that one of those munificent donations from time to time reported in other centres were made. The time was opportune, and the encouragement such a gift would be to the board in their contemplated scheme of development was yet to be realised.

Progress had been made with the acquisition of land surrounding the infirmary. By reason of the fact that the area was covered with buildings the cost was considerable, and £7,700 had already been expended in recent acquisitions.

The entire staff of the institution had responded well to many additional demands made upon their services, and their loyalty was cordially acknowledged. Many members of the past and present staff had rendered important services to the nation, and the long list of those who had received awards and distinction filled the governors with pride and satisfaction.

### Satisfactory Financial Position.

The estimated ordinary income was £45,760 and the expenditure £46,501, so that a small deficit of £740 was carried forward, which would be reduced if the estimated income were exceeded. £9,000 had been received in annual subscriptions, and the board were pleased that Mr. Alfred Corah's (treasurer's) personal appeal had been continuously successful. £3,000 would be set aside as a reserve fund for future contingencies. Donations amounted to £4,110, Hospital Sunday Fund £2,800, and Hospital Saturday Fund £17,500. Receipts from Government Departments for cost of maintenance of patients totalled £17,848. This income would undoubtedly show a decline in the following years, being in fact extraordinary income, uncertain and casual. The number of endowed beds and cots had been increased by seven, the capital sums received therefor amounting to £4,600. Extraordinary income was well maintained, legacies to the infirmary amounting to £4,775 and to the Children's Hospital £1,100. £2,000 of legacies to the infirmary was invested, and the balance would be appropriated towards the acquisition of property account.

The expenditure showed an increase of 15 per cent. on the past year, but it had been the policy of the board to purchase requirements well ahead of current needs, and the succeeding year's expenditure would show a corresponding decrease.

A new scale of salaries for sisters, nurses, and probationers came into operation on January 1, 1919, and would add £800 to the expenditure.

Appreciation was expressed at the way in which the nursing staff, entirely without outside assistance, had taken upon themselves the responsibility for the Christmas entertainments, which had given great pleasure to the patients.

Several names were added to the list of life governors in respect of donations. An apology for non-attendance was sent by Mr. H. W. Salmon, who, as a thanksgiving for the safety of his four sons in the war, enclosed a donation of £52 10s.

